# Editorial: Vibrionaceae Diversity, Multidrug Resistance and Management

**DOI:** 10.3389/fmicb.2018.00563

**Published:** 2018-03-26

**Authors:** Learn-Han Lee, Pendru Raghunath

**Affiliations:** ^1^Novel Bacteria and Drug Discovery Research Group, School of Pharmacy, Monash University Malaysia, Bandar Sunway, Malaysia; ^2^Center of Health Outcomes Research and Therapeutic Safety, School of Pharmaceutical Sciences, University of Phayao, Phayao, Thailand; ^3^Department of Microbiology, School of Medicine, Texila American University, Georgetown, Guyana

**Keywords:** *Vibrio* sp., multidrug resistance, pathogenesis, virulence, genome, prevalence

The genus *Vibrio* from Vibrionaceae family were originally isolated by an Italian physician, Filippo Pacini in 1854 (Thompson et al., 2004). Presently, the genus contains a total of 142 species which are marine originated and its taxonomy is continuously being revised due to the revelation of new species (Summer et al., 2001; Igbinosa and Okoh, 2008; Sawabe et al., 2013). Some of the *Vibrio* species are identified as foodborne pathogens causing illnesses upon consumption of contaminated seafood or water (Letchumanan et al., 2014; Law et al., 2015). The three *Vibrio* species primary for foodborne diseases are *Vibrio cholera*—associated from food contamination or transmission of infection from person to person, *Vibrio parahemolyticus*—associated with food contamination, and *Vibrio vulnificus*—associated with wound infection (Letchumanan et al.). Significant efforts have been applied to analyze the genetic as well as the molecular mechanisms underlying the infection actions of *Vibrio*.

This Research Topic brings together 19 articles that features the current discoveries concerning the biology of pathogenic *Vibrio* species covering from the epidemiology studies to discovery of antibiotic resistant strains in the environment which causes immense impact on the treatment and management of illnesses. The genome and non-antibiotic management articles in this research topic provides a deeper knowledge on *Vibrio*.

As mentioned in four of the articles, *Vibrio* sp. has a well diverse epidemiology. In Japan, this ecological pathogen can be found among seawaters of coral reefs in Ishigaki. The discoveries uncovered that Ishigaki coral reefs consisted of 22 known and 12 potential novel Vibrionaceae species. The abundance of *Vibrio* sp. had a significant positive correction with the rising seawater temperature. Amin et al. presented the results that would benefit in the coral conservation and future actions. Where else, in the neighboring nation Shanghai, China, *Vibrio parahaemolyticus* has been detected as a major pathogen for foodborne gastroenteritis cases. All the *V. parahaemolyticus* isolated from patients in Shanghai hospital exhibited T3SS1 and T3SS2 genes, 7/42 positive for *trh* gene and 37/42 positive for *tdh* gene. Further it was revealed that these clinical isolates expressed multidrug antibiotic resistance and carried resistance genes (Li et al.). Pathogenic *Vibrios* affecting bivalves in hatcheries has been reviewed by Dubert et al. This review article provided an insight on the *Vibrio* sp. affecting the hatcheries and proposed effective measures to curb the pathogens. These *Vibrios* can be managed through proper water treatments method in hatcheries, application of antibiotics, probiosis, quorum quenching, and phage therapy (Dubert et al.). The rarely unexplored *V. vulnificus* occurrence, healthcare burden and prevention were reviewed by Heng et al. *V. vulnificus* has been reported to be infecting in many countries worldwide and caused a huge healthcare burden. Efforts to treat *V. vulnificus* infections has hampered due to the emergence of multidrug resistant strains in the environment. It was suggested that appropriate choice of treatment would be cefotaxime and ceftriaxone since the first line of drugs may longer be applicable to treat *V. vulnificus* infections (Heng et al.).

A remarkable feature of *V. parahaemolyticus*'s ability of sensing human bile and regulate virulence reviewed by Letchumanan et al. The role of *V. parahaemolyticus* virulence starts upon ingestion of the bacterium and exposures to bile salts. Two encoded protein, VtrA and VtrC will cooperate and form a protein complex on the surface of the membrane of the bacterial cell. This complex structure then binds to bile salts and triggers the cell to produce toxins. Upon binding of bile salts to the VtrA/VtrC complex, the cytoplasmic DNA binding domain of VtrA is activated which in turn induces VtrB to activate, resulting in the T3SS2 expression. T3SS2 virulence is secreted thus causing illness to human. The information on bile-bacteria interaction pathway is important in order to develop drugs that can suppress bacterial virulence (Letchumanan et al.).

The emergence of multidrug resistant strains has become an international health crisis as causing significant threat to human well-being (WHO, 2014; Letchumanan et al., 2015). In Bangladesh, it was reported that *V. cholerae* O1 was resistant toward ciprofloxacin and azithromycin. These two antibiotics are currently used for prophylactic treatment of *V. cholerae* infections. The reduced susceptibility to ciprofloxacin and azithromycin is alarming for cholera-endemic countries of Asia and Africa (Rashed et al.). In Malaysia, *V. parahaemolyticus* strains isolated from shellfish was reported to be resistant against second and third lines of antibiotics. Majority of the strains were resistant toward 5 antibiotics out 14 antibiotics tested. These results are a real concern and warrants a continuous surveillance on the antibiotics used in the aquaculture settings (Letchumanan et al.). Similar antibiotic resistant pattern was discovered among *V. cholerae* non-O1/non-O139 collected from Haiti waters. The highest proportion of isolates were reported to be resistant against sulfonamide, amoxicillin, and trimethoprim-sulfamethoxazole. There was isolates exhibited resistance toward erythromycin and doxycycline, the two antibiotics used to treat cholerae in Haiti. The presences of *V. cholerae* non-O1/non-O139 with resistance pattern against clinically used antibiotics may cause public health threat (Baron et al.). Pathogenic *V. cholerae* and *V. vulnificus* with antimicrobial resistance patterns has been reported in Germany. The study further revealed that resistant toward carbapenems was observed in four environmental *V. cholerae* strains studied. Carbapenems are the last line of antibiotics for the treatment of *Vibrio* infections. The occurrence of carbapenemase producing *V. cholerae* in German coastal waters is of concern and highlights the need for monitoring the use of antibiotics (Bier et al.). In Senegal, antibiotic resistant *V. cholerae* O1 was isolated during a cholera outbreak. The isolates showed resistance pattern at least four antibiotics tested including trimethoprim-sulfamethoxazole, streptomycin, trimethoprim, and sulfamethoxazole (Sambe-Ba et al.).

Likewise, the genomics of *Vibrio navarrensis* has been explored via a phylogenetic relationship by performing a single nucleotide polymorphism (SNP) analysis. Such analyses provide an in-depth overview of unique molecular makers and virulence associated genes of *Vibrio navarrensis* (Schwartz et al.). Wang et al. has applied and study the use of multiple cross displacement implication combined with gold nanoparticle-based lateral flow biosensor for detection of *V. parahaemolyticus*. Based on the findings, the method was reported to be suitable for rapid screening of *V. parahaemolyticus* in clinical, food, and environmental samples (Wang et al.).

The management of *Vibrio* spp. using non-antibiotic approach has been well addressed in two review articles in this research topic. *Streptomyces* are soil living bacteria that has been widely recognized as industrially important microorganism due to its potential in producing various antagonistic compounds (e.g., anti-biofilm, anti-quorum sensing, and anti-virulence) against *Vibrio* pathogens. *Streptomyces* can be used as probiotic and incorporated in feeds in aquaculture to protect the livestock from pathogens. Despite the fact that there are promising outcomes introduced by past researchers on the use of Streptomyces as a probiotic in aquaculture, further extensive trials are needed to establish the probiotic nature of *Streptomyces* in disease prevention and development improvement of aquaculture animals (Tan et al.). Essentially, another review article discussed about the advantages of bacteriophages as a non-antibiotic agent. Bacteriophages as a non-antibiotic approach has gained popularity recently due to the increase emergence of antibiotic resistant bacteria worldwide. The bacteriophages have various advantages such as being host specific, environmental friendly, can be effortlessly found and isolated from ecological sources, and cost effective compared to antibiotics. These phages have been proven to control and inhibit virulence of *Vibrio* sp. isolated from both clinical and environmental. The recognition and safety accreditation by US Food and Drug Administration (FDA) on phages has open the doors for phages to be commercially manufactured for human application and consumption (Letchumanan et al.).

In summary, the collection of manuscripts provided in this Research Topic offers a comprehensive view on the prevalence and antibiotic resistance profiles of *Vibrio* sp. and as well as discussed, the management methods. This issue shows the significant progress and understanding in the control of pathogenic *Vibrio* sp. and provides insights to researchers worldwide about new and promising therapies.

## Author contributions

L-HL performed the literature search and writing of the manuscript. PR provided critical review and insight to improve the writing. The research project was conceptualized by L-HL.

### Conflict of interest statement

The authors declare that the research was conducted in the absence of any commercial or financial relationships that could be construed as a potential conflict of interest.
